# Gene Set Summarization using Large Language Models

**Published:** 2023-05-25

**Authors:** Marcin P. Joachimiak, J. Harry Caufield, Nomi L. Harris, Hyeongsik Kim, Christopher J. Mungall

**Affiliations:** 1 Biosystems Data Science Department, Environmental Genomics and Systems Biology Division, Lawrence Berkeley National Laboratory, 1 Cyclotron Road, Berkeley, CA 94720, USA; 2 Robert Bosch LLC, Sunnyvale, CA 94085, USA

## Abstract

Molecular biologists frequently interpret gene lists derived from high-throughput experiments and computational analysis. This is typically done as a statistical enrichment analysis that measures the over- or under-representation of biological function terms associated with genes or their properties, based on curated assertions from a knowledge base (KB) such as the Gene Ontology (GO). Interpreting gene lists can also be framed as a textual summarization task, enabling the use of Large Language Models (LLMs), potentially utilizing scientific texts directly and avoiding reliance on a KB.

We developed SPINDOCTOR (Structured Prompt Interpolation of Natural Language Descriptions of Controlled Terms for Ontology Reporting), a method that uses GPT models to perform gene set function summarization as a complement to standard enrichment analysis. This method can use different sources of gene functional information: (1) structured text derived from curated ontological KB annotations, (2) ontology-free narrative gene summaries, or (3) direct model retrieval.

We demonstrate that these methods are able to generate plausible and biologically valid summary GO term lists for gene sets. However, GPT-based approaches are unable to deliver reliable scores or p-values and often return terms that are not statistically significant. Crucially, these methods were rarely able to recapitulate the most precise and informative term from standard enrichment, likely due to an inability to generalize and reason using an ontology. Results are highly nondeterministic, with minor variations in prompt resulting in radically different term lists. Our results show that at this point, LLM-based methods are unsuitable as a replacement for standard term enrichment analysis and that manual curation of ontological assertions remains necessary.

## Introduction

Molecular biologists frequently need to interpret the results of experiments or investigations that result in lists of genes. These gene lists are then used to infer underlying mechanisms or phenomena. For example, a readout of genes expressed in cancer cells may be used to infer underlying signaling pathways, which in turn can suggest therapeutic approaches. Alternatively, a Genome-Wide Association Survey (GWAS) investigating a trait or disease may reveal correlations between variants in multiple genes and that trait.

Where only a few genes are involved, it may be possible for researchers to undertake an open-ended exploratory analysis to infer the underlying mechanism by researching each gene individually in the literature and databases, and summarizing these into a hypothesis. It may even be possible for a researcher to do this based on common knowledge of the genes involved in a pathway. However, even for small gene sets, this approach is time-consuming, subjective, and prone to bias. For larger gene sets, it is completely infeasible. Instead, researchers typically perform a gene set *enrichment* or *over-representation analysis*, in which curated *ontological annotations* of these genes are extracted and optionally compared against the annotations of the background set. These analyses make use of knowledge bases (KBs) that have two components: (1) an ontology, which provides a hierarchical logical organization of gene function descriptors; and (2) gene annotations, which associate genes with these descriptors. The ontology is used as part of the analysis, using reasoning to generalize to broader terms.

These enrichment analyses are part of the core fabric of molecular biology and biomedical research. The leading system is the Gene Ontology(Gene Ontology Consortium 2023), which provides ontological annotations of genes across the tree of life using over 40,000 descriptor terms. The GO is one of the most widely cited tools in the life sciences (Duck et al., 2016), and hundreds of tools implement GO enrichment analyses for a range of experimental modalities, from single cell analysis to GWAS. For example, a recent study measured gene expression at the single cell level in multiple populations in the vasculature of the human brain(Garcia et al., 2022). Each population was analyzed using GO, revealing functional roles of different cell subtypes, with implications for conditions involving cerebrovascular injury.

In addition to GO, other annotation systems can be used in enrichment analyses to reveal other salient aspects of the genes involved - for example, gene expression using an anatomy ontology(Bastian et al., 2021), or pathway database annotations(Fabregat et al., 2018).

More recently, instruction-based Large Language Models (LMMs) based on the Generative Pre-Trained (GPT) architecture(Brown et al., 2020) have attracted attention due to their highly general abilities on a wide range of text processing tasks, including information extraction, query construction, question answering, and text summarization. Instruction-based LLMs such as GPT-3 and successors are distinguished from the previous generation of LLM models such as BERT(Devlin et al., 2018) and BioBERT(Lee et al., 2019) by their ability to follow instructions in response to a prompt, and the ability to generalize from a small number of examples (few-shot or in-context learning). We have demonstrated that instruction-based LLMs can be used in conjunction with ontologies for KB and ontology extraction tasks(Harry Caufield et al., 2023), potentially as an aid to curation. Others have demonstrated the ability of LLMs such as GPT-4 to perform tasks such as annotation of single-cell sequencing data(Hou and Ji 2023).

Here we investigate the ability of GPTs to interpret lists of genes, such as those yielded by gene expression experiments and GWAS. We do this by reframing the task from one of *statistical enrichment* to a *text summarization* task, i.e. taking a larger text and condensing it into salient points. We devised a method that uses LLMs and configurable sources of gene descriptions to perform GPT-based summarization, taking as input a gene set and producing as output (1) a list of relevant terms, analogous to enriched terms in an over-representation analysis; and (2) a narrative summary that weaves together the different functions.

We explore three different summarization approaches. The first is purely generative, and relies solely on the massive corpus of documents ingested as training for the GPT model (which can be thought of as the “latent KB” of the model). The second makes use of narrative gene summaries, such as those authored by the curators of the RefSeq database(O’Leary et al., 2016). The third makes use of controlled textual summaries of GO annotations, such as those provided by the Alliance of Genome Resources (AGR) (Kishore et al., 2020). We evaluate all methods against standard enrichment analysis using the GO.

## Methods

### SPINDOCTOR: A novel method for gene set summarization using language models

**Figure 1:**
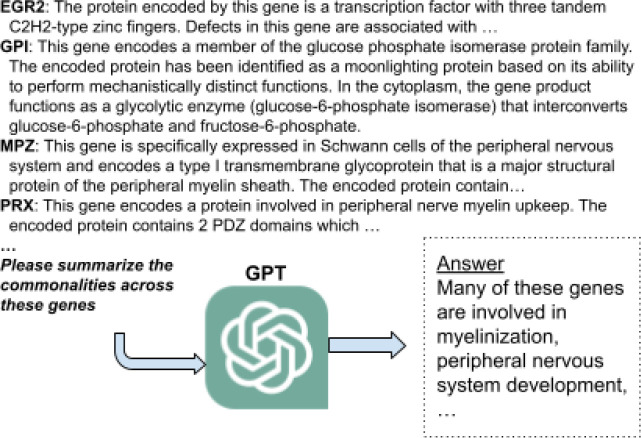
Illustration of gene set interpretation as a text summarization task. The genes of interest are included along with descriptions of their function (which could be as narrative text or controlled natural language), followed by a natural-language description of the task (“summarize commonalities”); this is called the “prompt”. The prompt is fed to a GPT LLM, which provides a functional summarization.

We created a method for summarizing gene sets using GPT models called SPINDOCTOR: *Structured Prompting Interpolating Narrative Descriptions Or Controlled Terms for Ontological Reporting*. This method takes as input a list of N genes *g*_*1*_*, g*_*2*_*, …, g*_*N*_ and produces two outputs: (1) a textual summarization of salient features of the gene set, and (2) a list of M ontology terms *t*_*1*_*, t*_*2*_*, …, t*_*M*_ from an ontology such as the GO. The method works by generating a *structured prompt*, containing textual summaries of genes from a list of sources; the prompt is also crafted to instruct the model to extract salient features of the gene sets (Fig. 1). The method is intended for large LLMs that have been fine-tuned on instruction-following tasks, such as GPT-3.5 models and successors (e.g. text-davinci-003, gpt-3.5-turbo, and gpt-4).

### Structured Prompt Generation

For each gene ID in the gene set, we query the Alliance of Genome Resources API(Alliance of Genome Resources Consortium, 2020) to retrieve (i) the gene symbol; (ii) a narrative gene description, aggregated from RefSeq; and (iii) automated gene descriptions(Kishore et al., 2020). Note that automated gene descriptions are in fact derived from curated ontological GO annotations; here “automated” refers to the ontology-to-text process rather than the process of generating the ontological annotations in the first place.

For each gene we generate a *description line* that is a concatenation of the gene symbol and the description separated by a colon character. For the narrative method, we use the narrative gene description, and for the ontological method we use the ontology term summaries. For the generative approach, we only provide the gene symbols.

We then generate a prompt using the Jinja template system(Ronacher 2008) with a standard template incorporating the gene description lines:

I will give you a list of {{ taxon }} genes together with descriptions of their functions.

Perform a term enrichment test on these genes.

i.e. tell me what the commonalities are in their function.

Make use of classification hierarchies when you do this.

Only report gene functions in common, not diseases.

e.g if gene1 is involved in “toe bone growth” and gene2 is involved in “finger morphogenesis”

then the term “digit development” would be enriched as represented by gene1 and gene2.

Only include terms that are statistically over-represented.

Also include a hypothesis of the underlying biological mechanism or pathway.

Provide results in the format

{{SUMMARY_KEYWORD}}: <high level summary>

{{MECHANISM_KEYWORD}}: <mechanism>

{{ENRICHED_TERMS_KEYWORD}}: <term1>; <term2>; <term3>

For the list of terms, be sure to use a semi-colon separator, and do not number the list. Always put the list of terms last, after mechanism, summary, or hypotheses.

Here are the gene summaries:

{GENE_DESCRIPTIONS}

Note the header includes an in-context directive specifically instructing the model to generalize over the gene sets, giving an example of how to do this.

### Token length limits and gene description truncation

One of the current limitations of LLMs is the number of tokens (roughly, the number of words) that can be provided as both input and output. If either gene lists are large, or the textual summaries of the genes are long, then the prompt will exceed the maximum token length (currently 4k for GPT-3.x models, and 8k or 32k for GPT-4).

In order to accommodate limits for number of tokens in different models we truncate the length of each gene description proportional to total number of tokens relative to maximum token length. We truncate from the end of the string, on the assumption that the text at the beginning is more informative.

Note this can result in quite substantial information loss, proportional to the number of genes in the input gene set. We record this as the truncation factor (TF); a TF of 1.0 reflects that the prompt was generated without truncation, while a TF of 0.25 indicates that only 25% of the original text could be used.

### Prompt Completion and Payload Parsing

Generated prompts are fed to the model via the OpenAI API. We use default configuration, with the lowest temperature setting (i.e. maximizing determinism). Results are cached to avoid expensive recomputation.

Our approach to prompt completion parsing reuses the method described in our SPIRES manuscript(Harry Caufield et al., 2023), in which the resulting GO terms are grounded using the Ontology Access Kit (OAK)(Mungall et al. 2023).

Note that our prompt asks for separate sections in the payload: a high level narrative summary, plus a list of terms. The narrative summary is not parsed and is presented to the user as-is. The string with the list of terms is split, and the resulting list is fed through the OAK annotator. Note this step assumes that the model yields descriptors that conform to the terminology of GO, using either the primary label or the synonym.

Our prompt explicitly avoids asking for GO identifiers or any other form of identifier. This is because we have previously observed that GPT-3.5 models frequently hallucinate “likely seeming” numeric identifiers.

### Implementation

The implementation is in Python as a part of the OntoGPT package (https://github.com/monarch-initiative/ontogpt)(Harry Caufield et al., 2023).

Access to a GPT model via an API such as the OpenAI API is required. However, for evaluation purposes, it is possible to use our cached completions.

Prompt completions are cached in a local sqlite3 database to avoid incurring charges by repeated requests of the same text. There is an interactive mode that bypasses API access and asks the user to copy the prompt into the web ChatGPT interface, and then copy the results back.

**Figure 3:**
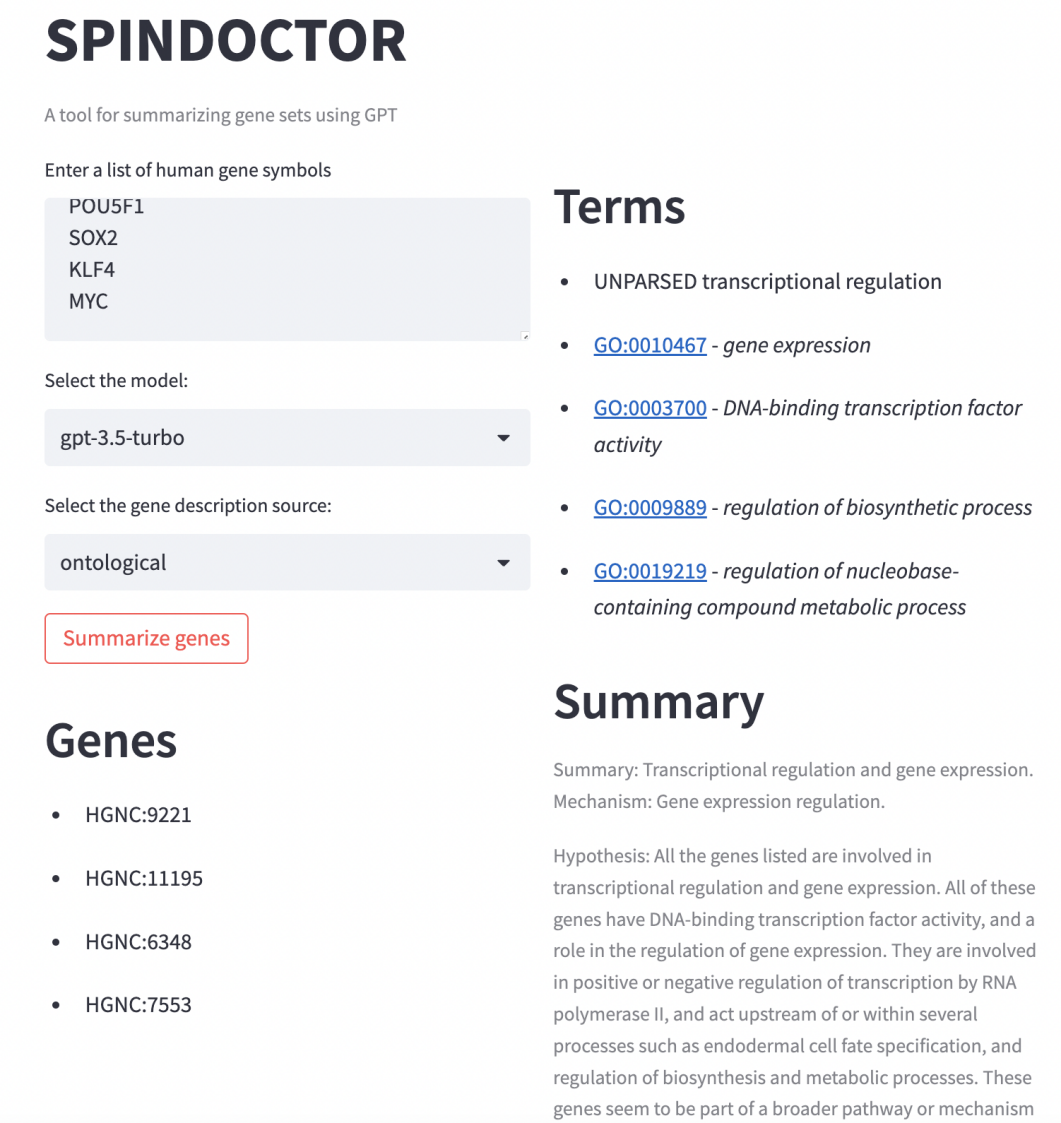
UI for SPINDOCTOR web application

We provide both a command line interface and a web application interface for SPINDOCTOR. The web application interface makes use of the streamlit framework, and currently must be executed locally. The web application UI is shown in Figure 3.

### Evaluation

There is no single agreed-upon approach to benchmarking enrichment analysis algorithms(Ballouz et al. 2017). For this study, we collected a set of 70 human gene sets for evaluation, from multiple sources including the literature, MSigDB(Dolgalev, n.d.), GeneWeaver(Baker et al., 2016), Human Phenotype Ontology Annotations(S. Köhler et al., 2021), disease to gene relationships from the Monarch Initiative(Shefchek et al. 2020), and, as a control, existing GO annotations. For the main evaluation all gene sets consist of human genes.

For each gene set, we generated an additional perturbed gene set simulating noise, where we dropped out 10% of genes and inserted random genes as replacements.

For each gene set and perturbed gene set we ran the three SPINDOCTOR methods with three different models, *gpt-3.5-turbo* and its predecessor *text-davinci-003*, as well as the newer gpt-4. We will refer to these as “turbo” and “davinci” and “gpt-4”in this manuscript. For gpt-4 we only completed a smaller analysis due to increased costs associated with this model.

We compare the results of SPINDOCTOR with standard gene set enrichment implemented in OAK, using hypergeometric tests and Bonferroni correction, treating this as the gold standard.

To measure and evaluate the performance of the GPT-based methods we use a number of different metrics, each of which can be applied to a particular GPT run on a particular gene set:

- **Proportion of significant terms**: how many of the GO terms returned were included in the statistically significant (adjusted p-value < 0.05) terms returned by standard enrichment analysis.
- **Has top term**? Was the top-scoring (most significant) term from standard enrichment included in the GPT set?
- **Number of GO terms in results**. Note this is not used directly to measure performance, but is included as an informative indicator of how “concise” the method is. This only measures terms from the prompt completion that could be grounded using the current GO vocabulary, so doesn’t include the unparsed terms below.
- **Number of unannotated terms**. i.e. GO terms that are neither directly nor indirectly (via propagation up the ontology graph) used to annotate any of the genes in the gene set. These terms may be a “hallucination” where the model invents a function, or may potentially reflect true gene function under-annotation.
- **Number of unparsed terms**. The number of terms returned in the enrichment list that cannot be parsed (grounded) to a GO term identifier using labels and synonyms in GO.

The results of the evaluation are available via Zenodo(Joachimiak 2023) and can be viewed through a Jupyter Notebook (https://github.com/monarch-initiative/ontogpt/blob/main/notebooks/Enrichment-Results-Analysis.ipynb).

## Results

### GPT methods yield valid but imprecise gene set summarizations from latent knowledge

We curated 70 gene lists and ran all methods on each gene set plus a perturbed copy. We tested two different gpt-3.5-based models, *text-davinci-003* (hereafter: *davinci*) and its successor *gpt-3.5-turbo* (hereafter: *turbo*). For each model, we tested the three sources of gene descriptions (ontological synopses, narrative synopses from RefSeq, and no synopsis). We evaluated the results of all methods across all gene sets. We deposited these results in a Zenodo-archived GitHub repository (https://github.com/monarch-initiative/enrichgpt-results/). The results are summarized in Table 2.

These results show that the newer turbo model outperformed its predecessor, davinci. Depending on the source of gene descriptions, the turbo model yielded statistically significant results between 60% and 65% of the time.

However, the model typically failed to return the top (most significant) term the majority of the time. This may be due to the inability of the model to generalize sets of terms using an ontology.

### Gene set summaries are biologically plausible in a way that disguises limitations

To gain a better understanding of how AI-based gene set summarization differs from standard statistical enrichment, we performed a qualitative assessment of the results of GPT summary derived term lists. When examined in isolation, these term lists were largely biologically plausible, valid (i.e. at least one gene that had the indicated function) across both different models (turbo vs davinci) and sources of gene description. However, when the results for a given gene set were compared across methods or compared to the gold standard statistical-ontological enrichment it revealed that results are often arbitrary and miss key terms that are often more informative.

**Figure 4:**
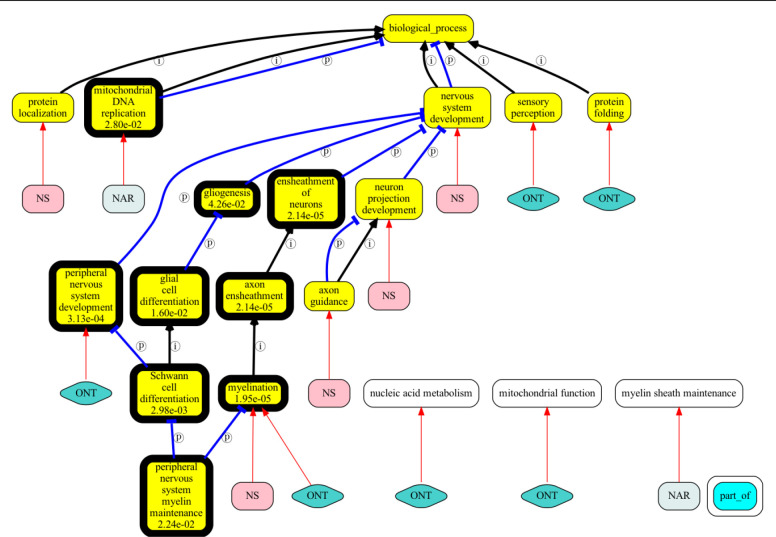
Superimposed results for summarization of sensory ataxia gene set (genes annotated to “Sensory ataxia”; HP:0010871). GO terms are in yellow, a bold border indicates significant (p-values shown in the text box). White terms are terms returned by a method that could not be grounded to a GO term identifier. ONT=ontological synopsis, NS=no synopsis, NAR=narrative synopsis.

This can be seen in Figure 4, which shows superimposed results (turbo only) for genes associated with the Human Phenotype Ontology term “Sensory ataxia” (HP:0010871; EGR2 NAGLU GPI DNAJC3 SH3TC2 TWNK PIEZO2 FLVCR1 MPZ PRX PMP22 KPNA3 POLG RNF170 AARS1).

We selected this gene set intentionally as an “easy” set with a clear underlying mechanism, to see what a good GPT result might look like. Genes implicated in Mendelian diseases are more likely to be studied and annotated. This particular phenotype of sensory ataxia has also been well studied with a large literature on underlying pathophysiological mechanisms(Lopriore et al. 2022).

Standard GO over-representation on this gene set yields “myelination” and “Schwann cell differentiation” as top (lowest p-value) hits. Figure 4 shows all terms found by all turbo methods against standard enrichment. The significant and gene-set relevant GO term “myelination” was found when using either ontologies as gene description or providing gene descriptions; however, when using narrative gene descriptions as a source, the string “myelin sheath maintenance” is returned, which essentially means the same thing, but automated methods do not ground this. Only the narrative based method found “mitochondrial DNA replication”. None of the GPT methods detected “Schwann cell differentiation”.

### Ontology annotations are the best summarization source when token limits are controlled for

We also investigated the extent to which description-based methods are penalized by prompt-completion token limits, by looking at the results for smaller gene sets, as these are less likely to be truncated. These results are shown in Table 3.

Ontology-based synopses perform best when descriptions are not truncated due to prompt token limits. For the full range of gene sets, ranging in size up to 200 genes, the best approach is to avoid providing a synopsis and instead rely on the model’s latent KB (66% of terms are significant, and 16% of the best terms are recapitulated). This is unsurprising, as truncating a description will eliminate much of the necessary information. When gene sets are smaller and truncation is low to non-existent, the ontological synopsis method emerges as the winner, with 61% of the most significant terms being recapitulated. While the truncation issue is a limitation, this is likely to be temporary with the newer gpt-4 based approach allowing for token windows up to 8 times larger.

Regardless of gene set size, ontological synopses also yielded a low level of unannotated GO terms (i.e terms not used to annotate any of the genes, or an ancestor of such term), from which we can speculate that this method is conducive to avoiding hallucination, although this may reflect it being too conservative, with unannotated terms actually reflecting valid informative summaries.

### GPT returns highly variable answers across different runs

To investigate the stability of LLM results we performed two runs for each model-method combination, where on the second run we made an insignificant syntactic change to the prompt (changing the end marker from 3 hashes to 3 equals symbols). We then measured the jaccard similarity of the term sets of each run (counting terms directly rather than using the ontology hierarchy). There was a very low level of consistency across runs, with the most consistent being turbo with ontological synopses. Consistency was over twice as high for turbo vs davinci.

### Larger models do not necessarily translate to better results

We performed a partial analysis of half of the gene sets (35) to evaluate whether the larger more expensive gpt-4 delivered advantageous performance. We were unable to perform a complete analysis due to the increased cost of running gpt-4 models.

Surprisingly, GPT-4 did not deliver major gains over the smaller turbo model, and in fact scored marginally worse overall.

### Generated narrative summaries are plausible but non-deterministic

We also examined the textual summaries produced by the turbo model with the 3 sources of gene descriptions. An example is provided in table 6, for the textual summaries and proposed mechanisms provided for the sensory ataxia gene set.

Overall, the qualitative summary of the gene sets appears to be plausible, although inconsistent in terms of being able to yield the most significant term. For example, in the above, the turbo model did not mention a key term, “myelination”.

We also investigated how consistent textual summaries are between runs. We calculated the cosine similarity of text embeddings of descriptions using the OpenAI *text-embedding-ada-002* model. Overall summaries generally varied quite widely, with turbo varying less widely than davinci.

### Hallucinations are rare to non-existent when summarizing human gene sets

A common problem with LLMs is the tendency to hallucinate(Ji et al. 2022). Previously we have observed that hallucinations are less problematic for knowledge-oriented *in-context* tasks(Caufield et al., 2023). Here we evaluate the extent to which LLMs hallucinate on a constrained gene set summarization task.

We took all turbo model results, and aggregated all unannotated terms for all GPT results. These represent potential hallucinations (i.e where the model fabricated a term for a gene set). We examined each instance and evaluated whether it was a reasonably valid term for that gene set. Here the criteria for reasonable validity was whether the term was descriptive for any gene in that gene set. We were unable to detect any true hallucinations - every term reported by GPT was in some way reasonably valid even if it did not meet the bar for GO annotation. These fell into different categories:

*Use of a term that has been obsoleted in the GO*: In this case, the model is likely recalling an older GO annotation to an obsolete term

*Regulation vs involved in*: for example, the Ehler Danlos Syndrome gene set summarization for includes the term *regulation of collagen metabolism*; the actual GO annotation was to a more precise *collage metabolism*.

*Alternate perspective*: a gene is annotated to a closely related term where the categorization is debatable. For example, the turbo model included the term “glucose transmembrane transporter activity” in the summary for the hallmark glycolysis gene set when given narrative gene set descriptions. Surprisingly, none of the GO annotations for any of the genes in the gene set included this term or a descendant of this term. However, one term in the gene set, SLC37A4, is annotated to glucose-6-phosphate transmembrane transporter. Formally this is not a glucose transporter as glucose-6-phosphate is a derivative of glucose. However, if GO were to make use of the CHEBI has-function-parent relationship when classifying GO terms then this gene would be classified as a glucose transmembrane transporter.

Note that although we were unable to detect any true hallucinations, it should be noted that many of terms given in summarization still fail to be statistically significant, as shown in Table 2.

Our hallucination analysis did not extend to the non-GO term summarizations. We observed that in some cases, these summaries included reports of p-values (even though we did not specifically ask for these), and while these looked plausible, they were in fact fabricated.

We also conducted experiments in which we explicitly asked for p-values to be included in the results, and as expected, these looked plausible but were in fact also fabricated. Thus when the requested task falls within general LLM capabilities (text summarization), hallucinations seem to be avoided, but when a request is made for something likely outside its capabilities (calculation of a statistical test), it will hallucinate.

We also conducted experiments where we spiked in gene descriptions from random other genes, to test whether the model was relying on gene symbols and its own latent KB of those genes, rather than the in-context information. For example, when running an analysis over the gene set for *canonical glycolysis*, we swapped out each gene description for a random gene description from a completely different gene set such as endocytosis. If the LLM were making use of its latent KB, then we might expect that the summary terms would still yield glycolysis terms, based on what the LLM “thinks” the genes do. In fact, regardless of whether the source was ontological synopses or narrative descriptions, the model used the descriptions, and summarized these, ignoring the gene symbols.

## Discussion

### Limitations of approach

We have developed and evaluated a method that performs gene set summarization using language models and configurable sources of gene synopses. While this method has some similarities to standard methods of gene set analyses, it is inherently more limited. Some of these limitations may be due to our own method, some may be inherent in the use of language models

- *No background sets*. Providing background sets of genes is crucial for accurate interpretation of results when not all genes were assayed. Providing descriptions of genes in the background set is challenging for LLMs due to constraints on the number of tokens that can be passed in a single prompt. Even if no gene descriptions are provided and we are making use of the LLM latent knowledge, the number of gene symbols may be too large. In future as newer models and techniques reduce token constraints it may be feasible to incorporate background genes.
- *Lack of statistics*. Standard methods of interpreting gene sets provide some statistical interpretation of the results, whether this is a p-value, or a probability in the case of model-based methods(Bauer et al. 2010). In contrast, language model based approaches rely on patterns in language. Although some have claimed that mathematical reasoning is an emergent ability of LLMs(Wei et al. 2022), we were unable to find a purely LLM-based way to generate reliable meaningful statistics for results, although this may change in the future. Of course, it is possible to implement a hybrid approach whereby the LLM hands terms off to a dedicated engine that implements the calculation, but this would only be possible for ontological annotation sources, so at this stage there is no real benefit to using an LLM at all.
- *Inherent non-determinism.* A current feature of LLMs is that output is highly non-deterministic, with minor variations in prompt resulting in sometimes massively different outputs. For a text summarization task this is not necessarily a problem, as there are many equally valid ways to summarize a task. But this becomes problematic when we try and apply summarization to interpreting scientific results, where we want to reduce arbitrariness and increase repeatability. One possibility here is to run the model multiple times and calculate some statistics over the results. We did not attempt to evaluate this here, in part due to the costs the repeated runs would incur, but this may be a promising avenue for future research
- *Inputs are unordered gene sets, not ranked lists*. Our method takes as input an unordered set of genes, similar to standard over-representation analysis. Many enrichment tools such as the Panther enrichment tool used by the GO Consortium(Mi et al. 2019) allow for rankings within the gene sets, applying the appropriate statistical test. We did not investigate the ability of LLMs to make use of ranked inputs. Including some kind of qualitative weights may be successful but we believe that as stated above using the appropriate statistical measure is likely outside current capabilities

Additionally our analysis has certain limitations. Evaluating and comparing gene set enrichment methods is challenging due to a lack of gold standards and agreed upon metrics. Previous approaches to evaluation include calculating mutual coverage of gene sets(Hung et al. 2012). We include a mutual coverage jaccard score in our full Jupyter notebook analysis. However, this is not a good method for evaluating text summarization, which is a different task. With a standard enrichment analysis, significance scores can be calculated for all terms in an ontology, but for text summarization the model selects only a small subset of relevant terms.

### Language models are not a shortcut to manual curation

Gene set enrichment and over-representation analyses rely on high-quality curated KBs such as the GO or Reactome. The use of AI and massive LLMs may seem like an opportunity to bypass curation and use information either from selected textual summaries or from a massive corpus of training data. However, this would be a serious mistake. First it is necessary to acknowledge that these LLMs almost certainly making heavy use of the curated content of these KBs. Information from the GO is replicated in multiple places, from encyclopedic resources such as Wikipedia through to major genomics knowledge portals such as the UniProt and NCBI Gene interfaces, and GO enrichment analyses are commonplace in the literature. Annotations are also frequently stored in repositories such as GitHub, which are included in LLM training sets. The power of LLMs to make use of this information seemingly intelligently is indeed remarkable, but this is all derived from highly curated content. This content needs to be constantly updated in light of new scientific knowledge, otherwise the quality of gene set enrichment results decreases significantly(Green & Karp, 2006; Tomczak et al., 2018; Wadi et al., 2016). Furthermore, our results show that when token length is controlled for, the best results are obtained using textual representations of ontological annotations; in this scenario, the LLM is essentially regurgitating existing annotations and does not provide any shortcut to curation. Using LLMs as a justification for bypassing curation would be severely misguided, would result in worse results over time, and is fundamentally misguided as investment in curation is minimal compared with the costs of performing the underlying experiments, with curation costs accounting for less than 0.01% of the whole (Karp, 2016).

Furthermore, we were unable to get LLM approaches to perform the same kinds of ontology-based generalizations we see with standard enrichment analyses, resulting overall in lower precision and key informative terms being missed in results. Additionally, results are highly non-deterministic, with minor prompt variations resulting in different term lists each run. Thus using a LLM may on the surface at first seem to deliver relevant and plausible results, the user may be unaware that the results are an arbitrary subset of possible results, and that they may be missing crucial information.

### Future Directions

Our methods described here employ zero-shot learning, with a small in-context example of how to generalize in a similar manner to the ontological generalization employed in standard term enrichment. It is possible that fine tuning could improve the ability to generalize sets of terms, or even to improve the relevancy and significance of these terms.

Our methods did not make use of the conversational abilities of LLMs, as exhibited by ChatGPT. The user has no opportunity to refine responses, or to interrogate results in finer grained detail. We envision future possibilities in which the user is able to enter a dialog, with LLM wrappers able to transparently interact with multiple different biological KBs as exhibited in the GeneGPT system (Jin et al. 2023).

### Further research is required on narrative outputs of LLM-based gene set summarization

Our methods and study focused on using LLM-based methods to generate GO term based summaries of experiments from underlying gene sets, analogous to standard term enrichment. We also demonstrated the ability to create narrative summaries of these gene sets, and even to provide mechanistic explanations of underlying biological processes. However, we did not attempt to evaluate this content, beyond demonstrating that this also frequently changed from run to run. Fully evaluating narrative output is much more challenging, as such an evaluation would be subjective and would itself require NLP techniques to automate, with attendant dangers of circularity.

However, while we were not able to systematically evaluate the quality of these narratives, we found many to sound plausible and even compelling. It is important to study this further, as there may be a temptation to use LLMs to “tell stories” about their data. This could be risky and highly problematic, due to well-known issues such as hallucinations and bias. We were not able to detect hallucination and bias in the term summarization tasks, but it is important to note that this is a highly constrained task with strong in-context cues; even here we are unable to guarantee the absence of these problems, and we were easily able to induce hallucinations by asking for something the model is unable to deliver (computed p-values).

When we move from summarization in the form of controlled term lists to more open-ended summarization tasks, such as generating narrative summaries, the dangers increase. These may be more nuanced than outright confabulation of results. LLMs have been shown to exhibit “behavior” such as sycophancy (telling the user what they want to hear) and sandbagging (detecting naivety on the part of the user and providing false information)(Bowman, 2023), all of which are potential risks when interpreting scientific data using background knowledge.

Although our evaluation ignored the textual summaries when parsing term lists, we noticed one occasion when the prompt completion provided additional misleading commentary at the end:

Note: These terms were statistically over-represented among the listed genes. The cytoskeletal reorganization was not statistically significant enough to be included. The underlying biological mechanism is likely related to the regulation of intracellular trafficking and signaling pathways, which are important for the maintenance of cellular homeostasis.

We know for a fact that the LLM did not perform a statistical test, despite what it may be telling us. However, it is true that cytoskeletal reorganization (closest match in GO is GO:0007010, “cytoskeletal organization”) is the function of some of the genes, but not enough to reach the level of statistical significance. The text above is therefore partially correct, but only by accident. However, results like this can easily ‘sandbag’ a researcher into over-interpreting or believing incorrect interpretations.

We can also easily imagine a scenario within a dialog-oriented scenario, a ‘sycophantic’ model is able to selectively give results that a researcher wants to hear. We were able to use the ChatGPT interface to prompt in such a way that almost any gene set can be interrogated in a way to satisfy an a-priori assumption, e.g. that the nervous system is involved.

This danger is compounded when we consider the fact that the leading models used in the kinds of higher-order instruction-based prompting demonstrated in this paper are not open, with essentially inscrutable training data(Bender et al. 2021), much of it derived from massive number of websites, likely including datasets such as the Colossal Clean Crawled Corpus (C4). Despite being cleaned, the C4 still includes significant content from web sites favoring white supremacist thought(Dodge et al. 2021; Inside the secret list of websites th...). The prospect of using models trained on this content to interpret human genetics data, bypassing curated content should be alarming.

Some of these dangers can be mitigated by moving towards open models where training sets are transparent, and by using curated trusted KB content via in-context cues. However, even with these measures, scientific interpretations derived from LLMs should not be used in place of standard KB enrichment systems.

## Conclusions

We investigated the ability of LLMs to perform gene set function summarization as compared to standard ontology-based gene set enrichment analyses. We compared different models and different sources of gene descriptions, and found that while the oldest models performed most poorly, the newer gpt-4 did not substantially outperform gpt-3.5-turbo. When token length limits are controlled for, using precise ontological descriptors derived from high quality manual curation and vetted propagation methods outperforms either narrative descriptions or relying on the models’ latent KB. When compared against standard enrichment, the results are typically plausible, relevant, and largely free of hallucination. However, the most precise and informative term is usually missed, likely reflecting the lack of an ability to generalize. The LLM approach also lacks statistical rigor, and the model is unable to natively provide p-values or reliable quantitative indicators of relevance of terms. Additionally, performance varies when genes are less well known, especially in the case of model organism genes. Results are also highly non-deterministic, with different terms provided on different runs.

Nevertheless, the results are striking given the relative newness of instruction-based LLMs, and illustrate powerful textual manipulation and in-context capabilities. The ability to generate narrative summaries alongside term lists is compelling, however, there are substantial risks of hallucination and bias associated with this approach. Our results underscore the need for high quality up to date curated KBs to assist with the interpretation of scientific data.

## Supplementary Material

Supplement 1

## Figures and Tables

**Figure 2:**
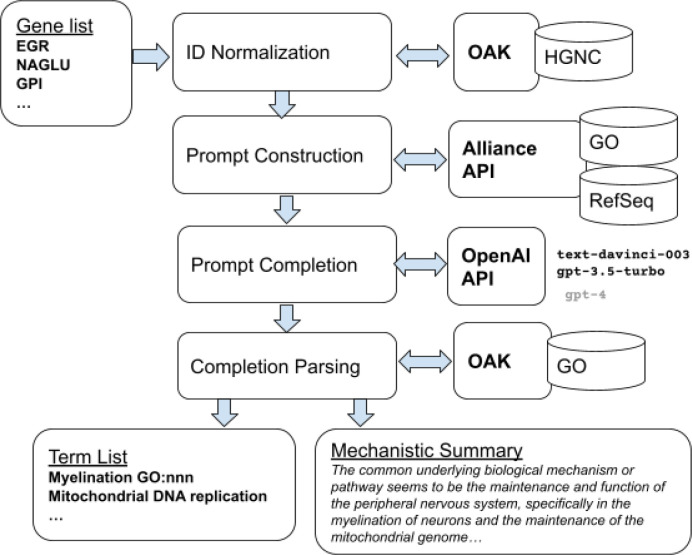
SPINDOCTOR pipeline. The user enters a list of genes, as symbols or identifiers. These are first normalized to identifiers (we use HGNC for human genes). An external source of information is queried to retrieve textual descriptions of genes, plus the canonical gene symbols; here we use the Alliance of Genome Resources API. These descriptions are used to construct a prompt, which is fed to the OpenAI API, which applies the prompt to a model. The resulting completion is parsed, with term lists normalized to identifiers using OAK (the Ontology Access Kit), yielding both a term list and a mechanistic summary.

**Table 1:** Different sources of gene descriptions/synopses as input for the SPINDOCTOR method. Note that when no explicit gene descriptions are supplied, GO annotations likely form part of the original GPT training corpus due to their ubiquity in many authoritative web resources.

Synopsis	Source of synopses	Explicit Curation
No synopsis	Underlying Language Model (“latent knowledge base”)	Indirect
Narrative synopsis	RefSeq Gene Summaries	Textual summary
Ontological synopsis	Alliance of Genome Resources (AGR) Automated Gene Descriptions	GO annotations

**Table 2:** Results of evaluation of all methods against the gold standard. Each column is the mean of the metric described in the methods section. Highest values for each metric are bolded. High scores for mean *proportion significant* and mean *has top term* reflect high-performing models. The average number of GO terms reflects how concise the method is, and the average number of unannotated terms (those not found in the annotated terms or their ancestors) reflect potential hallucination or under-annotation.

		proportion significant	has top term	num GO terms	num unannotated	num unparsed
**model**	**method**					
**gpt-3.5-turbo**	**narrative synopsis**	**0.657**	0.141	3.965	0.18	5.599
	**no synopsis**	0.64	**0.19**	4.954	0.225	6.884
	**ontological synopsis**	0.597	0.148	3.687	0.102	6.187
**text-davinci-003**	**narrative synopsis**	0.38	0.095	4.028	0.342	11.901
	**no synopsis**	0.436	0.085	3.461	0.285	10.018
	**ontological synopsis**	0.309	0.099	**6.915**	**0.408**	**13.623**

**Table 3:** Results on gene sets of size 75 or less. Here the ontological synopsis method emerges as the winner among GPT based methods.

		proportion significant	has top term	num GO terms	num unannotated	num unparsed
**model**	**method**					
**gpt-3.5-turbo**	**narrative synopsis**	0.602	0.163	3.043	0.228	4.935
	**no synopsis**	0.574	0.196	4.326	0.326	5.272
	**ontological synopsis**	**0.611**	**0.337**	3.902	0.12	5.348
**text-davinci-003**	**narrative synopsis**	0.326	0.12	3.348	0.326	11.337
	**no synopsis**	0.406	0.12	2.62	0.25	7.359
	**ontological synopsis**	0.338	0.217	**7.913**	**0.446**	**12.587**

**Table 4:** Summary statistics for Jaccard similarity of term lists when prompt is modified across all gene sets.

		count	mean	std	min	max
**model**	**method**					
**gpt-3.5-turbo**	**narrative_synop sis**	**142**	0.152	0.143	**0**	0.75
	**no_synopsis**	**142**	0.123	0.129	**0**	0.5
	**ontological_syn opsis**	**142**	**0.16**	**0.185**	**0**	**0.8**
**text-davinci-003**	**narrative_synop sis**	**142**	0.061	0.07	0	0.333
	**no_synopsis**	**142**	0.038	0.052	0	0.25
	**ontological_syn opsis**	**142**	0.084	0.095	0	0.5

**Table 5:** GPT-4 results, on a smaller subset of gene sets. On this set of gene sets, the best method is the combination of turbo plus no synopsis. However, when using ontology synopses as gene descriptions, gpt-4 delivers marginal improvements.

		proportion significant	has top term	num GO terms	num unannotated	num unparsed
**model**	**method**					
**gpt-3.5-turbo**	**narrative synopsis**	0.67	0.164	4.293	0.129	6.071
	**no synopsis**	**0.69**	**0.214**	5.136	0.15	7.279
	**ontological synopsis**	0.628	0.107	3.414	0.071	5.979
**gpt-4**	**narrative synopsis**	0.605	0.129	4.807	0.136	8.243
	**no synopsis**	0.675	0.157	5.336	0.057	8.171
	**ontological synopsis**	0.635	0.114	5.486	0.114	7.921
**text-davinci-003**	**narrative synopsis**	0.358	0.114	4.579	**0.379**	12.393
	**no synopsis**	0.427	0.093	3.457	0.264	11.314
	**ontological synopsis**	0.305	0.086	**6.929**	0.343	**14.85**

**Table 6:** Textual summary of the sensory ataxia gene set descriptions using the 3 different approaches.

Source	Summary	Mechanism
Ontological synopsis (turbo)	Genes are mostly involved in nerve function and diseases, especially Charcot-Marie-Tooth disease.	The genes are all involved in the process of myelination of nerve cells and other nervous system processes.
Narrative synopsis (turbo)	Genes are mostly involved in the maintenance and function of peripheral nerves in the human body. Mechanism: Myelination and proper function of Schwann cells in peripheral nerves.	These genes are involved in the proper formation and maintenance of myelin sheaths in peripheral nerves, as mutations in these genes result in various forms of peripheral neuropathies, such as Charcot-Marie-Tooth disease and Dejerine-Sottas syndrome. Dysfunction in the myelin sheath formation or maintenance can lead to disrupted nerve conduction and neuropathy.
No synopsis (turbo)	This set of genes is enriched for functions related to nervous system development and maintenance, as well as mitochondrial metabolism	These genes likely play roles in maintaining the proper functioning of neurons and supporting cells and in energy production in mitochondria.

**Table 6:** consistency of textual summaries between different runs.

		count	mean	std	min	max
**model**	**method**					
**gpt-3.5-turbo**	**RANDOM**	**142**	0.833	0.06	0.674	**1**
	**narrative_synopsis**	**142**	0.909	0.039	0.677	0.977
	**no_synopsis**	**142**	0.911	0.033	**0.807**	0.966
	**ontological_synopsis**	**142**	**0.917**	0.032	0.803	0.976
**text-davinci-003**	**narrative_synopsis**	**142**	0.877	0.087	0.67	**1**
	**no_synopsis**	**142**	0.83	**0.108**	0.663	**1**
	**ontological_synopsis**	**142**	0.868	0.093	0.676	0.957
