# Supplementary material for: Gene Set Summarization using Large Language Models
Source: ArXiv. 2023 May 25:arXiv:2305.13338v2. Preprint. [Version 2] (PMC10246080)
Supplement: Supplementary file 1 [file NIHPP2305.13338v2-supplement-1.pdf]

Supplementary Material

Language Models yield more generic terms

Sometimes term enrichment analysis will yield undesirably generic terms as results. For example, “regulation of biological process”, “binding”, “catalytic activity”.

We analyzed how the results of different methods fall into different GO subsets. GO subsets are manually created groupings of terms, usually defined to have broad coverage across the ontology hierarchy at a desired level of generality(Harris et al. 2004). Two subsets in particular are of interest, *goslim_generic*, which provides a set of high level core terms, and *goslim_agr*, which provides higher level groupings intended to drive displays such as the Alliance

ribbon(Alliance of Genome Resources Consortium, 2020). We used these to define two anti-subsets, consisting of the ancestors of these subsets that are not themselves in that subsets. These correspond to terms that are very generic, usually more generic than a biologist would find informative.

		anc_of_goslim_generic	anc_of_goslim_agr
model	method		
text-davinci-003	no_synopsis	**0.221**	0.117
	narrative_synopsis	0.193	**0.137**
N/A	standard	0.144	0.079
	rank_based	0.129	0.079
gpt-3.5-turbo	no_synopsis	0.121	0.051
text-davinci-003	ontological_synopsis	0.114	0.068
gpt-3.5-turbo	narrative_synopsis	0.112	0.06
	ontological_synopsis	0.084	0.057
N/A	random	0.058	0.037
	closure	0.045	0.019
	standard_no_ontology	0.035	0.013

Supp Table 1: proportion of terms that fall into two ultra-generic categories, sorted by goslim_agr ancestors.

		goslim_generic	anc_of_goslim_generic	goslim_agr	anc_of_goslim_agr	closure_of_goslim_generic	closure_of_goslim_agr
model	method						
N/A	closure	0.027	0.045	0.015	0.019	0.072	0.034
	random	0.1	0.058	0.075	0.037	0.158	0.112
	rank_based	0.183	0.129	0.151	0.079	0.312	0.23
	standard	0.048	0.144	0.036	0.079	0.192	0.115
	standard_no_ontology	0.063	0.035	0.036	0.013	0.098	0.049
gpt-3.5-turbo	narrative_synopsis	0.212	0.112	0.068	0.06	0.324	0.128
	no_synopsis	0.181	0.121	0.059	0.051	0.302	0.11

	**ontological_synopsis**	0.145	0.084	0.088	0.057	0.229	0.145
**text-davinci-003**	**narrative_synopsis**	0.232	0.193	**0.151**	**0.137**	0.425	**0.288**
	**no_synopsis**	**0.262**	**0.221**	0.127	0.117	**0.483**	0.243

Supp Table 2: analysis for goslim_generic and goslim_agr. The proportion of terms that fall into either this subset or ancestors (closure is the union of the subset plus ancestors)

Gene sets

source geneset	geneset_size
EDS	19
FA	19
HALLMARK_APICAL_JUNCTION	200
HALLMARK_GLYCOLYSIS	200
HALLMARK_HEDGEHOG_SIGNALING	36
HALLMARK_INTERFERON_GAMMA_RESPONSE	200
HALLMARK_MTORC1_SIGNALING	200
Yamanaka-TFs	4
amigo-example	36
bicluster_RNAseqDB_0	158
bicluster_RNAseqDB_1002	52
term-GO:0007212	28
endocytosis	16
go-postsynapse-calcium-transmembrane	33
go-reg-autophagy-pkra	17
hydrolase activity, hydrolyzing O-glycosyl compounds	91
meiosis I	54
mtorc1	200
peroxisome	8
progeria	4
sensory ataxia	15

Effects of Token Length Limit and Truncation

Both ontological and narrative synopsis methods are penalized by the maximum number of tokens allowed (4k for the GPT-3 series). We therefore sought to explore the effect of truncating gene descriptions on the results. We subsetted the results for gene sets less than length 75, as these are typically truncated less (see Fig X).

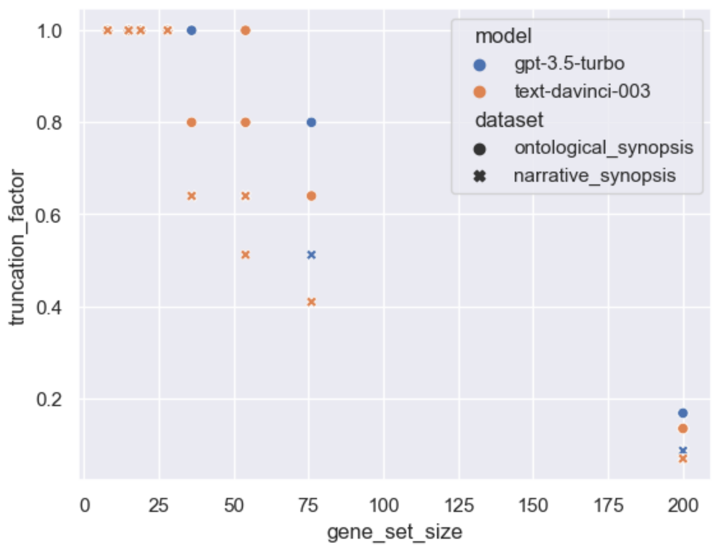

Figure: Relationship between gene set size and the proportion of synopsis text that is retained. Note that different models have different factors in calculating window size

Table: Rankings for smaller gene sets with fewer than 75 genes. This marginally boosts the performance of the LLM methods.

When gene descriptions are not truncated or only truncated minimally, the ontological synopsis method gains a further lead over the no-synopsis/narrative method. It seems that the performance of LLMs is not significantly degraded by truncating gene descriptions - whether this is due to the fact that information decreases as the text grows longer, or due to the LLM supplementing the lack of information with information from its own pre-trained corpus, we are unable to tell.
